# Transcranial Brain Stimulation Improves Cognition in Older Adults With Depression and Anxiety

**DOI:** 10.1093/geroni/igab046.2637

**Published:** 2021-12-17

**Authors:** Mathieu Figeys, Esther Kim, Ada Leung, Jim Raso, Hubert Kammerer, David Rawani, Glenrose Geriatric Research Team (Alberta Health Services)

**Affiliations:** 1 University of Alberta, Edmonton, Alberta, Canada; 3 Alberta Health Services, Edmonton, Alberta, Canada

## Abstract

Older adults admitted to hospital for rehabilitation often have some degree of concomitant cognitive impairment, which may be a barrier to optimizing rehabilitation approaches. Transcranial direct current stimulation (tDCS), a type of non-invasive brain stimulation, delivers a low electrical current across the brain. The neuromodulatory effects of tDCS can be of therapeutic benefit and has been shown to augment cognitive functions in both healthy and clinical populations. This study investigated the effects of tDCS on cognition in older adult inpatients with depression or anxiety. It was hypothesized that anodal tDCS over the left dorsolateral prefrontal cortex would increase cognitive performance compared to a placebo group. Twenty adults between 65 to 86 years of age admitted to the Glenrose Rehabilitation Hospital with underlying depression or anxiety were recruited. Anodal (n=10) or sham (n=10) tDCS stimulation was administered at 1.5mA over 20 minutes, for 10-15 sessions based on participant availability. Cognitive assessments were administered before and after the tDCS protocol. Anodal tDCS stimulation resulted in significant gains on the Symbol Digit Modality Test, Trail Making Test Part A, and Forward Digit Span. This study demonstrated a tDCS-invoked cognitive enhancement in the domains of attention, information processing speed, and short-term memory processes. With the rapidly ageing population, tDCS may be a potential therapeutic option for cognitive enhancement and may be beneficial in ageing-related cognitive-disorders including mild cognitive impairment and dementia.

